# Two Cases of Acute Rheumatism Successfully Treated upon Dr. Sutton's Plan

**Published:** 1814-04

**Authors:** H. S. Belcombe

**Affiliations:** Newcastle-under-Lyme


*For the Medical and Physical Journal.*

### *Two Cases of Acute Rheumatism successfully treated upon Dr. Sutton's Plan; by* Dr. H. S. Belcombe.

IN pointing out remedies serviceable to our fellow-creatures, the best reward to a liberal mind is the knowledge of their adoption. I am confident, therefore, Dr. Sutton will hear with pleasure, that amid various cases of acute rheumatism I have followed his directions with the earliest and happiest effects. But as facts are the soul of medicine, I shall give a short account of two of the severest cases.

Mrs. L., a respectable lady, of weak and delicate habit, was seized, on Saturday, Dec. 11th, with pain in the head and feet, which she attributed to exposure to cold in consequence of attendance on her sick husband. These complaints gradually grew worse, and on Dec. 14th, when I was called, I found every joint swollen, but no redness. She suffered excruciating pain on touch. Her heat was very great; tongue dry, white, with a red border; pulse 120, feeble; great thirst; bowels open, in consequence of aperients; perspiration, produced by antimonials, very copious, but afforded no relief. She complained likewise of pain in her chest and abdomen, attended with a very troublesome cough. I immediately removed some of the bed clothes, and ordered the cold camphire lotion—Acid. Sulph'. gr. xxx. Om. horâ. Pulv. Ipecac. c. gr. viij. nocte.

15th.—Night easier; pain somewhat abated; T. moist; complains of sickness; B. open; cough not so troublesome; urine scanty and high-colored. Rep. Lotio.

℞. Inf. Ros. ℥viij.
Potass. Nit. ʒj.

---

" The included matter (near perhaps to the nature of urine) was altogether incapable of suppuration, as it was saline and almost caustic; for very soon after its eruption it would corrode its vesicle, and burst out, of a color yellowish, livid, or black. Moreover, the surrounding circle was not always of the same appearance, although at first coming out it was continually inflamed.

" But this is highly observable, that sometimes these vesicles broke out without any other previous indications of infection, and, as I imagine, from the expeditious separation of the pestilential venom, and the sudden conquest of the distemper by a strong constitution. But whensoever the pain and heat of the part was so aggravated that no proper applications would assuage it, there was commonly danger of a mortification from so great a concourse of pestilential particles together; and once I remember a vesicle to change into a carbuncle, from the continued accession to it of fresh morbific poison."

Coch.

Coch. Mag. iij. Om. 3tia horâ.

Rep. Pulv. Nocte.

16th.—Pains in her joints much alleviated; could now use her hands; T. more moist; sickness gone; P. 96, rather full; breathing not very easy; no stool; thirst abated; urine more copious, and paler.

℞. Hyd. Sub. gr. iv.
Pulv. Jalap. gr. xiv.
Ammon. Carb. gr. viij.
Capiat ij. statim.*
Rep. Omnia alia Medicamina.

17th.—Considerably better; pain only in her feet; night easy; thirst gone; T. moist; P. 94, hard; pills had operated well; urine depositing copious red sediment; occasional heats, but no rigors.

19th.—Continues recovering. Complains only of pain in her back and shoulders, which seem to arise from weakness.

Omitte Omnia. Capiat Dec. Cinchonæ ter die.

She was soon following her usual avocations.

My next case was also that of a female, of a more robust habit; and here I was obliged to detract a considerable portion of blood. In every other respect the treatment was similar; and in less than a week I had the pleasure of restoring her to the free use of her limbs.

From these circumstances, connected with others, I am convinced that Dr. Sutton has pointed out a most valuable remedy for Rh. Acutus; and by this method its former agonies of weeks and months are resolved into a week's illness. It has also another merit, and that by no means an inconsiderable one—the being very grateful to the patient's feelings. While wishing to pay every tribute of respect to Dr. Haygarth's superior medical attainments, I still must confess myself sceptical with regard to the benefit derived from his practice in the disorder under consideration. I have seldom found it hasten a cure; not unfrequently, I have thought, protracted it. Till now I have generally preferred the willow bark (*salix latifolia*), which appears to me to sit more lightly on the stomach, and may, I think, be given in cases where we should hesitate to administer the cinchona.

*Newcastle-under-Lyme*, H. S. BELCOMBE, M.D.

*February*, 1814.

---

* The author has omitted to say in what form this prescription was to be compounded; but from the next day's report, we suppose he intended pills; still the number is not mentioned, nor the ingredients which might enable the compounder to make them up. We always correct errors when it is possible, but in this case it is impracticable.—Editors.

